# The Characteristics of the Influenza Virus Epidemic Around the SARS-CoV-2 Epidemic Period in the Pudong New Area of Shanghai

**DOI:** 10.1007/s44197-024-00194-9

**Published:** 2024-02-21

**Authors:** Ge Zhang, Anran Zhang, Li Zhang, Aiqin Zhu, Zhongjie Li, Weiping Zhu, Wenbiao Hu, Chuchu Ye

**Affiliations:** 1 School of Public Health, Dali University, Yunnan, China; 2 Shanghai Pudong New Area Center for Disease Control and Prevention, Shanghai, China; 3 Shanghai Mental Health Center, Shanghai Jiaotong University School of Medicine, Shanghai, China; 4 School of Population Medicine and Public Health, Chinese Academy of Medical Sciences and Peking Union Medical College, Beijing, China; 5 School of Public Health and Social Work, Institute of Health and Biomedical Innovation, Queensland University of Technology, Brisbane, Australia

**Keywords:** Subtype/lineage of influenza, SARS-CoV-2, Characteristics of influenza virus, COVID-19

## Abstract

**Objectives:**

The concurrent impact of COVID-19 and influenza on disease burden is a topic of great concern. This discussion delves into the epidemiological characteristics of seasonal influenza activity in Shanghai within the context of the SARS-CoV-2 epidemic.

**Methods:**

From 2017 to 2023, a total of 11,081 patients having influenza-like illness (ILI) were included in this study for influenza virus detection. Reverse transcription polymerase chain reaction (RT-PCR) assays were conducted according to standardised protocols to identify the types and subtypes of influenza viruses. The positivity rate of the influenza virus among the sampled ILI cases served as a surrogate measure for estimating various influenza seasonal characteristics, such as periodicity, duration, peak occurrences, and the prevalent subtypes or lineages. Epidemiological aspects across different years and age groups were subjected to comprehensive analysis. For categorical variables, the Chi-square test or Fisher's exact test was employed, as deemed appropriate.

**Results:**

A total of 1553 (14.0%) tested positive for influenza virus pathogens. The highest positivity rate for influenza was observed in adults aged 25–59 years (18.8%), while the lowest rate was recorded in children under 5 years (3.8%). The influenza circulation patterns in Shanghai were characterised: (1) 2 years exhibited semiannual periodicity (2017–2018, 2022–2023); (2) 3 years displayed annual periodicity (2018–2019, 2019–2020, and 2021–2022); and (3) during 2020–2021, epidemic periodicities of seasonal influenza viruses disappeared. In terms of influenza subtypes, four subtypes were identified during 2017–2018. In 2018–2019 and 2019–2020, A/H3N2, A/H1N1, and B/Victoria were circulating. Notably, one case of B/Victoria was detected in 2020–2021. The epidemic period of 2021–2022 was attributed to B/Victoria, and during 2022–2023, the influenza A virus was the dominant circulating strain.

**Conclusions:**

The seasonal epidemic period and the predominant subtype/lineage of influenza viruses around the SARS-CoV-2 epidemic period in Shanghai city are complex. This underscores the necessity for vigilant influenza control strategies amidst the backdrop of other respiratory virus pandemics.

**Supplementary Information:**

The online version contains supplementary material available at 10.1007/s44197-024-00194-9.

## Introduction

Influenza is currently one of the greatest public health threats [1]. The 1918 influenza A/H1N1 pandemic killed approximately 40 million people worldwide [2]. In 2009, the H1N1 virus caused 12,500 deaths in just 19 months globally [3]. Due to the extensive consequences of the influenza illness, it imposes a significant disease burden [4]. IFV-A circulated showed two peaks in January and August in southern China. IFV-B circulated from December to the following March in southern China [5].

On December 31, 2019, the Wuhan Municipal Health and Health Committee of Hubei Province, China issued the “Notice of Pneumonia in Wuhan” after 27 cases of pneumonia had been reported [6]. On 21 January 2020, the National Health Commission of the People's Republic of China announced 2019‐nCoV pneumonia as a category B infectious disease, and preventive and control measures were taken according to category A infectious diseases [7]. On 30 January 2020, the WHO declared the emergence of COVID-19 a public health emergency of international concern [8]. To control the COVID-19 pandemic, China has implemented a series of non-pharmaceutical interventions (NPIs) against COVID-19 since 2020 [9]. NPIs are aimed at reducing SARS-CoV-2 transmission and are likely to impact the epidemiology of other respiratory viruses [10]. In December 2022, the National Health Commission of the People's Republic of China announced that 2019‐nCoV pneumonia preventive and control measures were taken according to category B infectious diseases [11]. Research shows that multiple respiratory viruses can concurrently or sequentially infect the respiratory tract and lead to virus‒virus interactions [12]. Our aim of this study was to analyse and compare the epidemiological characteristics of influenza during the COVID-19 pandemic in Pudong New Area, Shanghai. We aimed to provide guidance for the development of more targeted vaccination strategies.

## Methods

### Influenza Virus Detection

The study was conducted in Pudong New Area, the largest district of Shanghai, which is one of the most developed international cities in the world. Shanghai is in eastern China (121.81° E, 31.14° N) [13]. Influenza-like illness (ILI) surveillance data were collected from sentinel hospitals. All data were provided by the Pudong New Area Center for Disease Control and Prevention (Pudong CDC). ILI was defined as body temperature ≥ 38 °C, accompanied by sore throat or cough [14]. In each sentinel hospital, nasopharyngeal swabs were collected from 5 or 20 ILI cases for influenza virus testing, resulting in an average of 10–40 specimens per hospital per surveillance week. Samples were stored at 4–8 °C and sent to the laboratory at the Pudong CDC for testing within 24 h. Reverse transcription PCR (RT-PCR) assays were performed to identify the types/subtypes of influenza virus, following standard protocols [15]. A surveillance year was defined as the period ranging from week 23 of 1 year (approximately the week of June 1) to week 22 of the following year (approximately the week of May 31), based on influenza surveillance data. The surveillance week started on Monday and ended on Sunday [14]. The start of the influenza epidemic period was defined as the first week during which the positive rate of influenza virus testing was higher than 10% and remained above that level for at least four consecutive weeks, and the end of an influenza epidemic period was defined as the first week during which the positive rate was lower than 10% and remained at that level for at least four consecutive weeks [16].

### Influenza Virus Epidemic Periodicity

Semiannual periodicity refers to the occurrence of two distinct influenza epidemic periods within a year, each characterised by a single peak in influenza positivity rates. Annual periodicity, on the other hand, denotes a single annual influenza epidemic period with a singular peak in positivity rates. Conversely, the disappearance of epidemic periodicity signifies a year in which the conditions necessary for the emergence of an influenza epidemic period are not met.

### SARS-CoV-2 Detection

Individuals who tested positive for nucleic acid with a threshold of 'CT ≤ 35' were classified as COVID-19 patients. There was no SARS-CoV-2 outbreak prior to January 2020. The period from January 2020 to December 2022 was characterised as the phase of SARS-CoV-2 pandemic control. Post-December 2022 was designated as the period marked by a lack of control over the SARS-CoV-2 pandemic.

### Data Analysis

We analysed data from sampled ILI patients from June 6, 2017, to May 31, 2023; the collected data included sex, age, date of illness onset and laboratory testing results. The positive rate was calculated by dividing the number of samples positive for influenza virus by the total number of samples tested. The age-specific influenza positive rate by subtype/lineage was calculated as the number of sentinel specimens testing positive for each subtype/lineage (numerator) among the ILI cases recruited for specimen collection (denominator) in each corresponding age group [17].

Data were analysed using R 4.3.0 (R Core Team, R: A language and environment for statistical computing. R Foundation for Statistical Computing, Vienna, Austria). The Chi-square test or Fisher’s exact test was used for categorical variables as appropriate. A *p* value of < 0.05 was considered statistically significant.

## Results

### Overall Characteristics of Influenza Virus Surveillance

From 2017 to 2023, 11,081 ILI patients were enrolled for influenza virus surveillance in this study; 5600 (50.5%) were males, and 5481 (49.5%) were females. The median age was 19 years [interquartile range (IQR): 7–34 years]. A total of 1553 (14.0%) ILI patient specimens tested positive for influenza virus pathogens, among which the positive rate of A/H1N1 was 3.3% and that of A/H3N2 was 5.6%; the positive rate of B/Yamagata was 1.2% and that of B/Victoria was 3.9%. The highest positive rate (23.5%) occurred in the 2017–2018 surveillance year, and the lowest rate (0.1%) occurred in the 2020–2021 year (Table 1 and 2).

**Table 1:** Demographic characteristics of influenza-like illness (ILI) cases from two sentinel surveillance sites in PuDong, Shanghai, 2017–2023

Characteristics	2017–2018	2018–2019	2019–2020	2020–2021	2021–2022	2022–2023	Overall
No. of patients	2134	2102	1591	1827	1698	1729	11,081
*Sex, n (%)*							
M	1048 (49.1)	1040 (49.5)	749 (47.1)	946 (51.8)	941 (55.4)	876 (50.7)	5600 (50.5)
F	1086 (50.9)	1062 (50.5)	842 (52.9)	881 (48.2)	757 (44.6)	853 (49.3)	5481 (49.5)
Age, year, median (IQR)	17 (7.34)	17 (7.31)	13 (7.31)	14 (6.33)	15 (7.32)	31 (16.49)	19 (7.34)
Age group (years), n (%)*							
0–4	265 (12.4)	242 (11.5)	250 (15.7)	332 (18.2)	230 (13.5)	86 (5.0)	1405 (12.7)
5–14	750 (35.1)	764 (36.3)	573 (36.0)	607 (33.2)	603 (35.5)	314 (18.2)	3611 (32.6)
15–24	238 (11.2)	257 (12.2)	159 (10.0)	171 (9.4)	215 (12.7)	253 (14.6)	1293 (11.7)
25–59	758 (35.5)	734 (34.9)	549 (34.5)	573 (31.4)	535 (31.5)	775 (44.8)	3924 (35.4)
60+	123 (5.8)	105 (5.0)	60 (3.8)	144 (7.9)	115 (6.8)	301 (17.4)	848 (7.7)

*Some columns do not add up to 100% because of rounding

**Table 2:** Age-specific influenza positive rates in different surveillance years, PuDong, Shanghai, 2017–2023

Age group (years), ILI% (*n*1/*n*2)	2017–2018	2018–2019	2019–2020	2020–2021	2021–2022	2022–2023	Overall
0–4	10.9 (29/265)	5.4 (13/242)	3.2 (8/250)	0.0 (0/332)	0.4 (1/230)	3.5 (3/86)	3.8 (54/1405)
5–14	14.8 (111/750)	15.6 (119/764)	18.2 (104/573)	0 (0/607)	5.8 (35/603)	6.1 (19/314)	10.7 (388/3611)
15–24	28.2 (67/238)	15.6 (40/257)	19.5 (31/159)	0 (0/171)	17.2 (37/215)	24.1 (61/253)	18.3 (236/1293)
25–59	32.7 (248/758)	16.8 (123/734)	20.8 (114/549)	0.2 (1/573)	12.5 (67/535)	24.0 (186/775)	18.8 (739/3924)
60+	37.4 (46/123)	14.3 (15/105)	16.7 (10/60)	0 (0/144)	4.3 (5/115)	19.9 (60/301)	16.0 (136/848)
Overall	23.5 (501/2134)	14.7 (310/2102)	16.8 (267/1591)	0.1 (1/1827)	8.5 (145/1698)	19.0 (329/1729)	14.0 (1553/11081)

*n1* number of influenza-positive cases, *n2* number of ILI patients tested

### Influenza Virus Activity Among Age Groups

During the six study years, the influenza-positive rate varied by age group (*p* < 0.001), and the overall influenza-positive rate was highest among aged 25–59 years (18.8%), followed by those aged 15–24 years (18.3%), and the lowest rate among children aged less than 5 years (3.8%) (Table 2).

During the 6-year surveillance period, the predominant influenza subtypes varied among different age groups. In the 2017–2018 surveillance, A/H3N2 was the predominant subtype across all age groups. In the 2021–2022 surveillance, B/Victoria emerged as the predominant subtype in all age groups. Interestingly, during the 2018–2019 surveillance, except for the 5–24-year age group where B/Victoria was predominant, the other age groups exhibited A/H1N1 as the dominant subtype. In the 2019–2020 surveillance, A/H3N2 was the predominant subtype among individuals aged less than 14 years, while individuals aged over 15 years showed a predominance of B/Victoria. Among individuals over 60 years, A/H1N1 was the predominant subtype, whereas the other age groups demonstrated a predominance of A/H3N2 during the 2018–2019 surveillance period.

For influenza virus, A/H3N2 was the predominant subtype in the 5 age groups. For influenza A virus, the positive rate of A/H3N2 was higher than the positive rate of A/H1N1 among the 5 age groups. For influenza B virus, the positive rate of B/Victoria peaked in patients 15–24 years (6.1%), and B/Victoria was rarely detected among people 0–4 years (0.3%). However, B/Yamagata peaked in 15–29 years (1.7%) and was rarely detected among people 0–4 years (0.1%) (Table S1).

### Characteristics of Influenza Epidemics

**Fig. 1:**
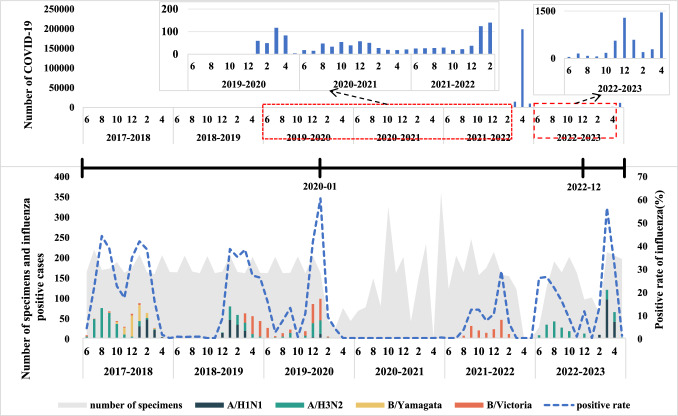
**a** COVID-19 epidemic duration times in Pudong, 2017–2023; **b** influenza epidemic duration times in Pudong, 2017–2023. Temporal trends of influenza virus activity by subtype/lineage. The shaded area represents the total number of specimens tested. The dark blue dotted line indicates the positive rate of influence

During the 6-year surveillance period, six epidemic periodicities of seasonal influenza viruses were identified: (1) 2 years had semiannual periodicity (2017–2018, 2022–2023); (2) 3 years had annual periodicity (2018–2019, 2019–2020, and 2021–2022); and (3) during 2020–2021, epidemic periodicities of influenza viruses disappeared. The majority of annual periodicity occurred in winter (Table 3 and Fig. 1).

**Table 3:** Characteristics of seasonal influenza virus epidemics in PuDong, Shanghai, 2017–2023

Characteristics	2017–2018		2018–2019	2019–2020	2020–2021	2021–2022	2022–2023	
Epidemic period	2017/7/31–2017/9/26	2017/12/19–2018/3/6	2018/12/25––2019/6/26	2019/11/26–2020/2/6	NA	2021/9/14–2022/2/8	2022/7/14–2022/9/15	2023/2/21–2023/5/3
Epidemic season	Summer	Winter	Winter\Spring	Winter	NA	Autumn\Winter	Summer	Spring
Start time (week number)	31	51	52	48	NA	37	26	8
End time (week number)	42	10	23	6	NA	6	37	16
Duration (number of weeks)	12	12	24	11	NA	22	12	9
Peak time (week number)	35	52	4	4	NA	3	32	14
Predominant subtypes	A/H3N2	B/Yamagata	B/Victoria	B/Victoria	NA	B/Victoria	A/H3N2	A/H1N1

In 2017–2018, two epidemic periods were identified in summer and winter. The influenza epidemic in the summer peaked at week 35, and the positive rate in that week was 65.0% (13/20). The peak in the winter occurred in week 5, in which the positive rate was 46.3% (19/41). In 2022–2023, two epidemic periods were identified in summer and spring. The influenza epidemic in the summer season peaked at week 32, and the positive rate in that week was 41.7% (15/36). The peak in the winter occurred in week 14, in which the positive rate was 63.0% (29/46). During weeks 26–37 of the 2022–2023 surveillance year, the influenza positivity rate did not meet the criteria for defining an influenza epidemic period. However, there was an observed trend of influenza activity from July to October 2022. Weeks 26–37 were defined as a special influenza epidemic period in the 2022–2023 surveillance year. Regarding year-round periodicity in three consecutive surveillance years, 2018–2019, 2019–2020 and 2021–2022, the influenza epidemic started in different seasons. In the 2018–2019 surveillance year, the epidemic started in autumn, and the epidemic ended in summer. The duration of epidemics is longer than that of other surveillance years. The peak in the spring seasons occurred in week 5, in which the positive rate was 52.6% (10/19). In 2019–2020, the epidemic started in winter, while in the 2021–2022 surveillance year, the epidemic started in autumn. However, the end times were the same at week 6 in the surveillance years 2019–2020 and 2021–2022. The peaks of the epidemic period were similar: weeks 2 and 3 in the surveillance years 2019–2020 and 2021–2022, respectively, and the positive rates at the peak times were 60.0% (24/40) and 34.1% (14/41), respectively.

**Fig. 2:**
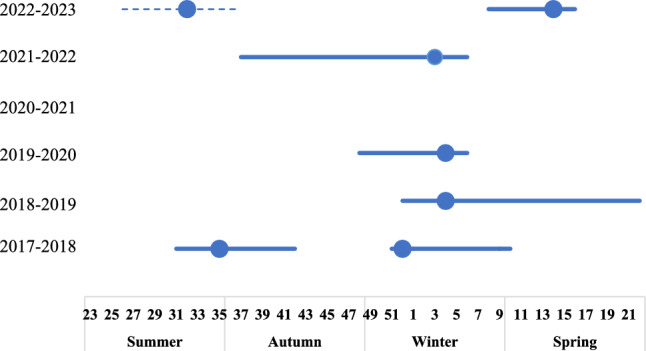
Influenza epidemic duration and peak times in Pudong, 2017–2023, using the positive rate as a proxy. The dark blue dot indicates the peak time. The dotted line shows the special epidemic period

During the 2017–2018 surveillance year, four distinct influenza subtypes were detected. A/H3N2, A/H1N1, and B/Victoria circulated during both the 2018–2019 and 2019–2020 surveillance years. In the 2020–2021 surveillance year, one case of B/Victoria was identified. The epidemic period in the 2021–2022 surveillance year was attributed to B/Victoria. In the 2022–2023 surveillance year, influenza A virus predominated (Table 3 and Fig. 2).

### Characteristics of COVID-19 Epidemiology

From January 2020 to February 2022, the monthly count of confirmed COVID-19 cases remained below 200. In April 2022, the number of confirmed COVID-19 cases peaked at 198,649. Non-pharmaceutical interventions were lifted in December 2022, and in May 2023, the number of confirmed COVID-19 cases reached its highest level at 11,746 [29].

## Discussion

This study holds novel significance in elucidating the dynamics of influenza virus activity in Shanghai before and during the 2019 SARS-CoV-2 pandemic. This underscores the intricate nature of influenza activity.In the past 3 years (2020–2022), the surveillance of influenza has been significantly impeded as a result of non-pharmacological interventions implemented for COVID-19. Consequently, the occurrence of influenza has exhibited a distinctive epidemic pattern during the aforementioned period of 2020–2022. Importantly, ILI can also be caused by other respiratory pathogens, such as respiratory syncytial virus (RSV) and adenovirus; however, we did not conduct diagnostic tests for these pathogens. The lower influenza positivity rate observed among children may be attributed to a higher prevalence of RSV infections within this age group [18]. Our findings indicated the possibility of two distinct influenza circulation patterns in Pudong,annual and semiannual epidemics,observed during the surveillance period spanning from 2017 to 2023. Notably, a relatively high proportion of A/H3N2 cases was observed across all age groups in this study, aligning with influenza surveillance outcomes reported in other subtropical regions of China [19].

During the study period, the highest influenza-positive rate in the tested ILI patients was found in 25–59 years (18.8%), and the lowest rate was found in children aged younger than 5 years (3.8%). These findings aligned with the outcomes of recent investigations conducted in China [19].

The 6-year surveillance period exhibited unique characteristics in each year. The influenza activity in 2017–2018 aligned with the typical patterns of an influenza epidemic. Notably, previous studies have reported distinct seasonality in China: Regions with latitudes greater than 33°N experience winter epidemics, areas with latitudes less than 27°N witness peak activity in spring, and provinces situated at intermediate latitudes exhibit semi-annual epidemic cycles [20]. In 2018–2019, the summer influenza epidemic period disappeared. However, the winter influenza epidemic period continued to the next surveillance year. Notably, B/Yamagata has not been detected since the 2018–2019 surveillance year. Previous research has suggested the possibility of B/Yamagata's potential extinction [21]. The peak positivity rate observed in the 2019–2020 season was the highest throughout the 6-year surveillance period and exhibited a rapid subsequent decline. Notably, similar patterns of peak positivity rates were also documented in Singapore and the United States [22, 23]. The reason for the rapid decline in the peak positive rate is Chinese measures of NPIs for COVID-19. During the 2020–2021 surveillance year, one case of B/Victoria was detected. According to the US CDC official website influenza report, from 20 weeks in 2020 to 20 weeks in 2021, the positive rate of influenza was less than 1.0% [24].

During the SARS-CoV-2 pandemic, as well as in the periods spanning from March to May in 2020–2021 and 2022, sample sizes exhibited instability due to NPIs. Furthermore, in the 2021–2022 season, a winter epidemic was solely attributed to B/Victoria, likely influenced by these interventions. After the policy of “zero-COVID-19” was changed in December 2022 [25], the peak positive rate, with influenza A as the major contributor, was delayed by approximately 2 months. Studies have indicated that various respiratory viruses can concurrently or sequentially infect the respiratory tract, resulting in potential interactions between these viruses [12, 26]. The results from a prospective cohort study of the UK reported that the emergence of SARS-CoV-2 was associated with substantial reductions in the circulation of seasonal respiratory viruses and large differences in the characteristics of viral-associated disease [27].

## Conclusions

In summary, our findings underscore the variability in influenza positivity rates and subtypes across different age groups, seasons, and NPIs. It is evident that the spread of influenza viruses is influenced by a multitude of factors [28]. We elucidate disparities in the prevalence of influenza viruses in Pudong, Shanghai, both before and after the onset of the COVID-19 pandemic. This serves as a reminder that even during concurrent respiratory virus pandemics, the importance of influenza prevention and control should not be underestimated. Consistently administering annual seasonal influenza vaccines remains the most efficacious measure for averting seasonal influenza and mitigating its potential risks.

### Supplementary Information

Below is the link to the electronic supplementary material.

Supplementary file1 (XLSX 10 KB)

## Data Availability

All data and statistical code to reproduce the results in the manuscript are available from the corresponding author upon reasonable request.

## Abbreviations

ILI: Influenza-like illness
RT‒PCR: Reverse transcription polymerase chain reaction
NPIs: Non-pharmaceutical interventions
RSV: Respiratory syncytial virus
IQR: Interquartile range
PDCDC: Pudong New Area Center for Disease Control and Prevention
